# Maternal residential proximity to chlorinated solvent emissions and birth defects in offspring: a case–control study

**DOI:** 10.1186/1476-069X-13-96

**Published:** 2014-11-19

**Authors:** Jean D Brender, Mayura U Shinde, F Benjamin Zhan, Xi Gong, Peter H Langlois

**Affiliations:** Department of Epidemiology & Biostatistics, Texas A&M Health Science Center School of Public Health, College Station, TX 77843-1266 USA; Department of Geography, Texas Center for Geographic Information Science, Texas State University, San Marcos, TX 78666 USA; Texas Department of State Health Services, Birth Defects Epidemiology and Surveillance Branch, MC 1964, PO Box 149347, Austin, TX 78714-9347 USA

**Keywords:** Air pollution, Chlorinated solvents, Congenital heart defects, Limb deficiency defects, Neural tube defects, Oral cleft defects

## Abstract

**Background:**

Some studies have noted an association between maternal occupational exposures to chlorinated solvents and birth defects in offspring, but data are lacking on the potential impact of industrial air emissions of these solvents on birth defects.

**Methods:**

With data from the Texas Birth Defects Registry for births occurring in 1996–2008, we examined the relation between maternal residential proximity to industrial air releases of chlorinated solvents and birth defects in offspring of 60,613 case-mothers and 244,927 control-mothers. Maternal residential exposures to solvent emissions were estimated with metrics that took into account residential distances to industrial sources and annual amounts of chemicals released. Logistic regression was used to generate odds ratios and 95% confidence intervals for the associations between residential proximity to emissions of 14 chlorinated solvents and selected birth defects, including neural tube, oral cleft, limb deficiency, and congenital heart defects. All risk estimates were adjusted for year of delivery and maternal age, education, race/ethnicity, and public health region of residence.

**Results:**

Relative to exposure risk values of 0, neural tube defects were associated with maternal residential exposures (exposure risk values >0) to several types of chlorinated solvents, most notably carbon tetrachloride (adjusted odds ratio [aOR] 1.42, 95% confidence interval [CI] 1.09, 1.86); chloroform (aOR 1.40, 95% CI 1.04, 1.87); ethyl chloride (aOR 1.39, 95% CI 1.08, 1.79); 1,1,2-trichloroethane (aOR 1.56, 95% CI 1.11, 2.18); and 1,2,3-trichloropropane (aOR 1.49, 95% CI 1.08, 2.06). Significant associations were also noted between a few chlorinated solvents and oral cleft, limb deficiency, and congenital heart defects. We observed stronger associations between some emissions and neural tube, oral cleft, and heart defects in offspring of mothers 35 years or older, such as spina bifida with carbon tetrachloride (aOR 2.49, 95% CI 1.09, 5.72), cleft palate with 1,2-dichloroethane (aOR 1.93, 95% 1.05, 3.54), cleft lip with or without cleft palate with ethyl chloride (aOR 1.81, 95% CI 1.06, 3.07), and obstructive heart defects with trichloroethylene (aOR 1.43, 95% CI 1.08, 1.88).

**Conclusions:**

These findings suggest that maternal residential proximity to industrial emissions of chlorinated solvents might be associated with selected birth defects in offspring, especially among older mothers.

## Background

Oxidative stress has been suggested as a mechanism by which some teratogens cause birth defects [1], and several chlorinated solvents, such as carbon tetrachloride, chloroform, methylene chloride, and trichloroethylene, have been identified as inducing oxidative stress through giving rise to reactive oxygen species [2]. These compounds have been widely used as solvents for industrial processes, such as metal degreasing and dry cleaning, and for the production of pharmaceuticals, pesticides, adhesives and refrigerants [3]. Findings from several experimental studies of animal models have indicated significantly increased prevalence of various congenital malformations, including exencephaly, musculoskeletal defects and cardiac defects with exposures to some chlorinated solvents [4–8]. These findings were not corroborated, however, by other investigators with experimental studies of rodents exposed to methylene chloride, methyl chloroform, perchloroethylene, and trichloroethylene [9, 10].

Several epidemiologic studies have examined the relation between maternal occupational exposure to various chlorinated solvents and birth defects in offspring [11–14] with associations noted between these compounds and cleft lip with or without cleft palate [11], spina bifida [12], limb defects [13], and heart defects [14]. Very limited information has been published on the relation between residential proximity to industrial air releases of chlorinated solvents and birth defects. Yauck et al. [15] examined whether living near industrial facilities in Wisconsin (USA) with trichloroethylene (TCE) emissions increased the risk of congenital heart defects in offspring. Mothers who lived within 1.32 miles of the TCE-emitting facilities had a three-fold excess risk of congenital heart defects, but this association was restricted to mothers aged 38 years and older. Two studies conducted in Texas (USA) [16, 17] found no elevated risk of oral cleft or conotruncal heart defects among offspring born to mothers who lived within 1 mile of any industrial facility with reported air emissions of solvents. Increased odds ratios for isolated oral clefts, especially for cleft palate, were noted among offspring to mothers 35 years or older in the Texas study population, although the 95% confidence intervals for the odds ratios were compatible with the null. In the same study population, residential proximity to industrial facilities with solvent emissions was modestly associated with neural tube defects, and a stronger association was noted in offspring of older mothers [18]. The aforementioned studies of potential maternal ambient air exposures to chlorinated solvents and birth defects did not take into account residential proximity to multiple sites nor the amounts of chemicals released from each of the facilities. Furthermore, the study populations tended to be small, thereby limiting examination of associations of birth defects with specific chemicals by maternal age.

In the present study, we examined whether maternal residential proximity to industrial air emissions of chlorinated solvents was associated with neural tube, oral cleft, limb deficiency, and congenital heart defects in offspring. We accounted for residential proximity to multiple facilities and the annual amounts of specific chlorinated solvents released from each. We also examined whether older maternal age modified associations between residential proximity to chlorinated solvent emissions and birth defects with one of the largest study populations to date.

## Methods

### Study population

Live births, fetal deaths, and induced terminations with selected congenital malformations, including neural tube defects, oral cleft defects, limb deficiencies, and congenital heart defects, were identified from the Texas Birth Defects Registry (TBDR) for births occurring during 1996–2008. The Registry used active surveillance in which trained staff reviewed medical records from hospital, birth centers, and midwifery locations. TBDR covered all cases that were live births, spontaneous fetal deaths (reportable in Texas if 350 grams or more or length of gestation 20 weeks or greater), and pregnancy terminations. Cases were selected in the study if they were delivered between 1996 through 2008, had a maternal address in Texas at time of delivery, and had a diagnosis of one or more of the selected birth defects. The majority of case deliveries were live births (97.1%) with 1.4% classified as spontaneous fetal deaths, 1.5% induced terminations of pregnancy, and 0.1% unspecified fetal deaths. Live birth and fetal death cases were linked to their respective birth or fetal death certificates that contained information about maternal residential address at the time of delivery and demographic characteristics.

Birth defects were further classified as either isolated or not isolated. Isolated defects were defined as those having a single major defect alone or together with one or more of a list of minor defects used in the National Birth Defects Prevention Study [19].

Control-births, defined as live births without any recorded birth defects, were randomly selected from live birth certificate data obtained from the Center for Health Statistics at the Texas Department of State Health Services. These births were frequency matched to cases by year of delivery (1996–2008) and public health service region (11 regions) in which the case-mothers resided at the time of delivery. We frequency matched birth defect cases and controls by public health region because between 1996 and 1999, active surveillance of birth defect cases did not occur statewide, but was confined to certain regions, including 2 regions in 1996, 6 regions in 1997, and 8 regions in 1998. These regions range from 12,060 (Region 5) to 61,456 square miles (Regions 9/10 combined).

ESRI ArcGIS 8.3 ArcMap 9.3 was used to geocode the maternal addresses obtained from live birth and fetal death certificates. A total of 86.7% of the maternal addresses for case-mothers and 87.2% for those of control-mothers were successfully geocoded to street level.

### Linkage to industrial air emissions of chlorinated solvents

Data regarding air emissions from Texas industrial facilities were obtained from the U.S. Environmental Protection Agency Toxic Release Inventory (TRI) program. The online TRI databases contain names of facilities required to report under section 313 of the Emergency Planning and Community Right-to-Know Act and include information about location, reporting year, chemicals released, and estimated pounds per year released into various environmental media, such as air and water bodies. For the present project, industrial air releases of 14 chlorinated solvents were identified for study, including carbon tetrachloride; chloroform; 1,1-dichloroethane; 1,2-dichloroethane; 1,2-dichloroethylene; ethyl chloride; methyl chloroform; methylene chloride; perchloroethylene; propylene dichloride; tetrachloroethane; 1,1,2-trichloroethane, trichloroethylene; and 1,2,3-trichloropropane.

Based on the addresses available in the USEPA TRI databases, we geocoded the TRI facilities in three steps. The addresses were first geocoded using Centrus Geocoder for ArcGIS. Then, the TRI addresses that were not geocoded in the first step were geocoded by the default geocoding tools from ArcGIS 10.0 using the Census 2000 street map as the reference layer. Finally, the remaining TRI addresses that were not successfully geocoded using the first two methods were geocoded with Google map geocoding API. Only TRI addresses geocoded to the premise level were considered successful matches. Over the 13 year period of 1996–2008, approximately 90% of the industrial facilities, on average, were successfully geocoded.

To estimate potential intensities of various chlorinated solvent air releases in the vicinity of maternal residences, a modified version of the Emission Weighted Proximity Model (EWPM) was used [20]. This model takes into consideration emissions from all sources within a 10-km effective threshold potentially affecting a person at a specific location and the amounts of chemical released from each source during a given time period [20]. The formula for calculating the combined quantity of air pollutant *θ* at location *i* from all emission sources is given by Expression (1).
1

where *A*^*θ*^_*i*_ is the estimated quantity of air pollutant *θ* at location *i* from all emission sources *j (j = 1, 2, ……, m)* within the threshold distance. This quantity was used as a proxy to represent the exposure risk value of a person at location *i* to air pollutant *θ*; *m* is the number of emission sources relevant to a person at location *i* in the area in question; *E*^*θ*^_*ij*_ is the emission rate of air pollutant *θ* from any emission source *j* that is within the effective threshold distance related to location *i*; *T*^*θ*^_*ij*_ is the duration of emission of air pollutant *θ* from emission source *j*; *k*^*θ*^ is effective threshold distance beyond which air pollutant *θ* is considered to have no harm to an individual and is determined by the physical and photochemistry characteristics of the pollutant in question [19]; and *D*_*ij*_ is the distance between location *i* and location of *j.* Based on Expression (1), an exposure risk value for each of the 14 chlorinated solvents was assigned to case- and control-mothers based on their residence location at the time of delivery. The TRI facilities and maternal addresses were linked through the EWPM. Given the effective threshold distance, only the facilities within the distance of a maternal address were considered to have potential harm on that person.

### Data analyses

The EWPM exposure risk values were categorized into two groups (exposure risk values for a given solvent equaling zero or greater than zero) and four or seven levels (exposure risk values at zero and greater than zero divided into three or six equal groups) based on the control-mothers distribution of scores. In the analyses with more than two categories of exposure risk values, the number of categories used (4 or 7) depended on the numbers of cases available. We used logistic regression to examine the relation (odds ratio [OR] and 95% confidence interval [CI]) between maternal residential exposures to chlorinated solvents and neural tube defects, oral clefts, limb deficiencies, and selected heart defects in offspring. The lowest level of exposure risk value served as the referent group for all analyses. In the analyses of odds ratios associated with varying intensities of exposure risk values, the Wald statistic was used to test for significance of linear trends. To reduce potential confounding, the ORs were adjusted for the year of delivery (1996–2008), maternal age (<20, 20–24, 25–29, 30–34, 35–39, >39 years), education (<12 years, 12 years, >12 years), race/ethnicity (white non-Hispanic, black non-Hispanic, Hispanic, other non-Hispanic), and public health region of residence (11 regions in Texas).

Because sufficient numbers of exposed cases were available, analyses of the association between chlorinated solvents and oral clefts were restricted to isolated defects. Analyses of the other types of birth defects included all identified cases. Heart defects were grouped into five major categories including conotruncal defects (truncus arteriosus, transposition of the great arteries, double outlet right ventricle, and Tetralogy of Fallot); obstructive heart defects (Ebstein anomaly, aortic valve stenosis, hypoplastic left heart syndrome, coarctation of the aorta, pulmonary artery atresia); septal defects (ventricular septal defect, atrial septal defect); atrioventricular septal defects (atrial septal defect primum, single common atrium, complete atrioventricular canal, endocardial cushion defect); and anomalous pulmonary venous return.

We also examined the effect of maternal age on the risk of selected birth defects associated with higher residential exposures to solvents. The EWPM exposure risk values as dichotomous variables (0, >0) were stratified by categories of maternal age (<35 years and 35 years or older) and age-specific ORs and respective 95% CIs were calculated. Additive and multiplicative interactions were assessed for the associations of birth defects with EWPM exposure risk values that appeared to vary by maternal age. Additive interaction was examined using a statistical program developed by Andersson et al. that estimated measures of relative excess risk due to interaction (RERI) and attributable proportion due to interaction (AP) [21]. If either or both measures differed from zero and their 95% CIs excluded 0, significant additive interaction was considered present. To assess multiplicative interaction, the product terms of EWPM risk values for a given solvent with maternal age were included in the logistic models and were considered significant if the p-value was less than 0.05.

## Results

Table 1 provides a comparison of case-mothers (of deliveries with neural tube defects, limb deficiencies, oral clefts, and congenital heart defects) and control mothers by demographic characteristics, delivery year, public health region of residence at the time of delivery, and geocoding status of residence. A total of 3245 cases with neural tube defects, 2406 cases with limb deficiencies, 7416 cases with oral clefts, 60154 cases with selected heart defects, and 280764 controls without major birth defects were available for the study. Compared with control-mothers, mothers of neural tube defect cases were more likely to be Hispanic and have less education, while mothers of babies with limb deficiencies and oral clefts were more likely to be non-Hispanic White. Mothers with babies with limb deficiencies and oral clefts were more likely than control-mothers to smoke during pregnancy, although smoking appeared underreported overall, given the low prevalence reported on the birth and fetal death certificates. Maternal residential street addresses were successfully geocoded to street level for 87.2%, 69.1%, 83.9%, 84.7%, and 87.4% respectively for control-births, and births with neural tube defects, limb deficiencies, oral cleft defects, and heart defects. Maternal addresses for neural tube defect cases were less likely to be geocoded because 21% of these deliveries were induced terminations for which maternal residential addresses were unavailable.

**Table 1:** **Selected maternal characteristics of birth defect cases and controls, Texas Birth Defects Registry, 1996-2008**

Characteristic	Controls (n = 280764)		Neural tube defects (n = 3245)		Limbs deficiencies (n = 2406)		Oral cleft defects (n = 7416)		Heart defects (n = 60154)	
	***n***	%	***n***	%	***n***	%	***n***	%	***n***	%
Race-ethnicity										
Non-Hispanic white	93425	33.3	956	31.7	890	38.3	2831	39.0	20434	34.2
Non-Hispanic black	27119	9.7	246	8.1	257	11.1	542	7.5	6144	10.3
Hispanic	150334	53.6	1749	58.0	1114	48.0	3617	49.8	31441	52.7
Others, non-Hispanic	9510	3.4	66	2.2	61	2.6	277	3.8	1670	2.8
Missing	376		228		84		149		465	
Education										
< High school	91641	32.9	998	39.2	732	32.9	2424	34.2	19523	33.1
High school	82138	29.5	785	30.9	691	31.0	2176	30.7	17448	29.5
>High school	104427	37.5	760	29.9	804	36.1	2481	35.0	22059	37.4
Missing	2558		702		179		335		1124	
Age at delivery (years)										
11-19	41376	14.7	462	15.2	379	16.3	1063	14.6	7973	13.3
20-24	79641	28.4	860	28.3	647	27.8	2014	27.7	15433	25.8
25-29	74994	26.7	823	27.1	609	26.2	1915	26.3	15145	25.3
30-34	55326	19.7	579	19.0	438	18.8	1378	18.9	12050	20.2
35-39	24487	8.7	248	8.2	184	7.9	689	9.5	6977	11.7
>39	4912	1.8	68	2.2	68	2.9	216	3.0	2193	3.7
Missing	28		205		81		141		383	
Public health service region										
1	9368	3.3	93	3.0	91	3.9	296	4.1	1957	3.3
2	5864	2.1	70	2.3	44	1.9	199	2.7	1219	2.0
3	76992	27.4	921	30.2	730	31.4	2092	28.7	16117	27.0
4	7080	2.5	106	3.5	82	3.5	285	3.9	1351	2.3
5	4640	1.7	48	1.6	48	2.1	161	2.2	950	1.6
6	50124	17.9	532	17.5	390	16.8	1395	19.2	10557	17.7
7	22544	8.0	259	8.5	230	9.9	723	9.9	4594	7.7
8	28740	10.2	314	10.3	296	12.7	778	10.7	6053	10.1
9	6932	2.5	84	2.8	60	2.6	202	2.8	1441	2.4
10	8576	3.1	131	4.3	84	3.6	309	4.3	1702	2.8
11	59904	21.3	487	16.0	272	11.7	839	11.5	13838	23.1
Missing	-		200		79		137		375	
Year of birth										
1996	6964	2.5	125	4.1	61	2.6	181	2.5	1444	2.4
1997	12648	4.5	170	5.6	102	4.4	355	4.9	2659	4.5
1998	16468	5.9	222	7.3	153	6.6	476	6.5	3398	5.7
1999	19096	6.8	287	9.4	186	8.0	575	7.9	3866	6.5
2000	20040	7.1	257	8.4	186	8.0	616	8.5	4092	6.9
2001	20588	7.3	232	7.6	204	8.8	605	8.3	4243	7.1
2002	21580	7.7	226	7.4	192	8.3	574	7.9	4566	7.6
2003	22980	8.2	232	7.6	192	8.3	579	8.0	4927	8.2
2004	25428	9.1	264	8.7	183	7.9	646	8.9	5468	9.2
2005	27464	9.8	248	8.1	211	9.1	672	9.2	5981	10.0
2006	27548	9.8	248	8.1	213	9.2	648	8.9	6004	10.0
2007	28828	10.3	266	8.7	228	9.8	665	9.1	6286	10.5
2008	31132	11.1	269	8.8	216	9.3	688	9.4	6846	11.4
Missing	-	-	199		79		136		374	
Smoking^a^										
No	230492	94.5	2061	94.9	1802	92.5	5684	92.2	48978	94.1
Yes	13338	5.5	111	5.1	145	7.5	479	7.8	3061	5.9
Missing	36934		1073		459		1253		8115	
Geocode accuracy										
9-Digit Zip	684	0.2	4	0.1	3	0.1	18	0.2	135	0.2
Manual	299	0.1	19	0.6	4	0.2	17	0.2	55	0.1
Street	244927	87.2	2241	69.1	2018	83.9	6282	84.7	52591	87.4
Zip	5955	2.1	42	1.3	39	1.6	156	2.1	1133	1.9
Not Geocoded	28899	10.3	267	8.2	197	8.2	686	9.3	5737	9.5
Missing	-	-	672	20.7	145	6.0	257	3.5	503	0.8

^a^Anytime during pregnancy.

Neural tube defects were significantly associated with maternal residential proximity to several types of chlorinated solvent air emissions (Table 2), most notably with carbon tetrachloride (adjusted odds ratio [aOR] 1.42, 95% CI 1.09, 1.86); chloroform (aOR 1.40, 95% CI 1.04, 1.87); 1,2 dichloroethane (aOR 1.28, 95% CI 1.01, 1.62); ethyl chloride (aOR 1.39, 95% CI 1.08, 1.79); methyl chloroform (aOR 1.29, 95% CI 1.01, 1.63); 1,1,2 trichloroethane (aOR 1.56, 95% CI 1.11, 1.28); and 1,2,3 trichloropropane (aOR 1.49, 95% CI 1.08, 2.06). Further examination of the neural tube defect phenotypes revealed that spina bifida, but not anencephaly, was associated with these chemicals. Spina bifida was also associated with maternal residential proximity to emissions of 1,1-dichloroethane (aOR 1.70, 95% CI 1.06, 2.71), 1,2 dichloroethylene (aOR 1.60, 95% CI 1.01, 2.53), and tetrachloroethane (aOR 1.78, 95% CI 1.12, 2.82).

**Table 2:** **Maternal residential proximity to air emissions of chlorinated solvents and neural tube defects, Texas, 1996-2008**

Type of chlorinated solvent	***Exposure risk value >0*** ^***a***^													
	Controls		Neural tube defect cases		Adjusted odds ratio ^b^(95% CI)		Anencephaly cases		Adjusted odds ratio ^b^(95% CI)		Spina bifida cases		Adjusted odds ratio ^b^(95% CI)	
	***n***	%	***n***	%			***n***	%			***n***	%		
Any type	63599	26.2	619	28.4	0.98	(0.88, 1.09)	173	29.3	1.04	(0.84, 1.27)	371	28.3	0.97	(0.85, 1.11)
Carbon tetrachloride	4568	1.9	62	2.8	1.42	(1.09, 1.86)	13	2.2	1.18	(0.66, 2.10)	44	3.4	1.58	(1.15, 2.19)
Chloroform	3985	1.6	52	2.4	1.40	(1.04, 1.87)	10	1.7	1.09	(0.57, 2.09)	37	2.8	1.55	(1.10, 2.20)
1,1-dichloroethane	1847	0.8	24	1.1	1.36	(0.90, 2.05)	5	0.8	1.13	(0.46, 2.77)	19	1.4	1.70	(1.06, 2.71)
1,2-dichloroethane	6637	2.7	85	3.9	1.28	(1.01, 1.62)	13	2.2	0.69	(0.39, 1.23)	65	5.0	1.64	(1.24, 2.16)
1,2-dichloroethylene	2026	0.8	25	1.1	1.26	(0.84, 1.89)	5	0.8	0.99	(0.40, 2.44)	20	1.5	1.60	(1.01, 2.53)
Ethyl chloride	5271	2.2	70	3.2	1.39	(1.08, 1.79)	16	2.7	1.28	(0.76, 2.15)	50	3.8	1.59	(1.18, 2.14)
Methyl chloroform	5294	2.2	79	3.6	1.29	(1.01, 1.63)	15	2.5	0.85	(0.50, 1.44)	57	4.3	1.56	(1.18, 2.07)
Methylene chloride	38448	15.9	401	18.4	1.03	(0.91, 1.16)	109	18.4	1.03	(0.82, 1.30)	240	18.3	1.02	(0.87, 1.18)
Perchloroethylene	29854	12.3	277	12.7	0.92	(0.81, 1.06)	78	13.2	0.95	(0.73, 1.22)	162	12.3	0.90	(0.75, 1.07)
Propylene dichloride	1518	0.6	16	0.7	1.15	(0.70, 1.90)	1	0.2	−^c^	−	14	1.1	1.58	(0.92, 2.72)
Tetrachloroethane	1955	0.8	23	1.1	1.31	(0.86, 2.00)	3	0.5	−^c^	−	20	1.5	1.78	(1.12, 2.82)
1,1,2-trichloroethane	2561	1.1	38	1.7	1.56	(1.11, 2.18)	6	1.0	0.97	(0.42, 2.21)	30	2.3	1.94	(1.32, 2.84)
Trichloroethylene	28547	11.8	274	12.6	0.95	(0.83, 1.09)	78	13.2	0.99	(0.76, 1.29)	163	12.4	0.94	(0.79, 1.12)
1,2,3-trichloropropane	2847	1.2	41	1.9	1.49	(1.08, 2.06)	8	1.4	1.15	(0.56, 2.36)	31	2.4	1.78	(1.22, 2.59)

^a^Exposure risk value based on residential proximity to source(s) of air emissions and estimated pounds of chemical emitted annually.

^b^Adjusted for birth year and maternal age, education, race/ethnicity, and public health region of residence (referent group: exposure risk value = 0).

^c^Odds ratios and respective 95% confidence intervals are not reported for analyses with less than five exposed cases.

With respect to chlorinated solvent emissions and oral clefts, a few positive associations were observed with cleft palate alone (Table 3). Propylene dichloride, in particular, was associated with cleft palate (aOR 1.77, 95% CI 1.05, 2.99).

**Table 3:** **Maternal residential proximity to air emissions of chlorinated solvents and isolated oral cleft defects, Texas, 1996-2008**

Type of chlorinated solvent	***Exposure risk value >0*** ^***a***^													
	Controls		Any oral cleft defect		Adjusted odds ratio ^b^(95% CI)		Cleft palate alone		Adjusted odds ratio ^b^(95% CI)		Cleft lip with or without cleft palate		Adjusted odds ratio ^b^(95% CI)	
	***n***	%	***n***	%			***n***	%			***n***	%		
Any type	63599	26.2	1036	26.8	0.99	(0.91, 1.07)	308	26.5	0.95	(0.82, 1.10)	729	27.0	1.00	(0.91, 1.10)
Carbon tetrachloride	4568	1.9	91	2.4	1.10	(0.88, 1.37)	32	2.8	1.25	(0.86, 1.80)	59	2.2	1.03	(0.79, 1.36)
Chloroform	3985	1.6	79	2.0	1.05	(0.83, 1.33)	25	2.1	1.03	(0.68, 1.56)	54	2.0	1.06	(0.80, 1.40)
1,1-dichloroethane	1847	0.8	33	0.9	1.00	(0.70, 1.42)	13	1.1	1.24	(0.71, 2.16)	20	0.7	0.89	(0.57, 1.40)
1,2-dichloroethane	6637	2.7	119	3.1	0.99	(0.81, 1.20)	45	4.1	1.10	(0.79, 1.54)	78	2.9	0.94	(0.74, 1.20)
1,2-dichloroethylene	2026	0.8	38	1.0	1.04	(0.75, 1.44)	14	1.2	1.19	(0.69, 2.04)	24	0.9	0.97	(0.64, 1.47)
Ethyl chloride	5271	2.2	107	2.8	1.10	(0.90, 1.34)	34	2.9	1.09	(0.77, 1.56)	73	2.7	1.11	(0.87, 1.41)
Methyl chloroform	5294	2.2	105	2.7	1.06	(0.87, 1.30)	30	2.6	0.99	(0.68, 1.45)	75	2.8	1.09	(0.86, 1.39)
Methylene chloride	38448	15.9	672	17.4	1.06	(0.97, 1.16)	193	16.6	0.99	(0.84, 1.17)	480	17.8	1.09	(0.98, 1.21)
Perchloroethylene	29854	12.3	462	12.0	0.98	(0.88, 1.08)	127	10.9	0.88	(0.72, 1.07)	335	12.4	1.02	(0.90, 1.15)
Propylene dichloride	1518	0.6	29	0.8	1.07	(0.74, 1.56)	15	1.3	1.77	(1.05, 2.99)	14	0.5	0.75	(0.44, 1.28)
Tetrachloroethane	1955	0.8	36	0.9	1.05	(0.75, 1.48)	17	1.5	1.61	(0.98, 2.64)	19	0.7	0.81	(0.51, 1.28)
1,1,2-trichloroethane	2561	1.1	54	1.4	1.16	(0.87, 1.53)	22	1.9	1.51	(0.97, 2.34)	32	1.2	1.00	(0.70, 1.43)
Trichloroethylene	28547	11.8	483	12.5	1.02	(0.92, 1.13)	144	12.4	1.00	(0.83, 1.21)	339	12.5	1.02	(0.90, 1.16)
1,2,3-trichloropropane	2847	1.2	61	1.6	1.16	(0.89, 1.51)	24	2.1	1.48	(0.97, 2.25)	37	1.4	1.03	(0.73, 1.44)

^a^Exposure risk value based on residential proximity to source(s) of air emissions and estimated pounds of chemical emitted annually.

^b^Adjusted for birth year and maternal age, education, race/ethnicity, and public health region of residence (referent group: exposure risk value = 0).

In analyses of congenital heart defects, only septal heart defects showed a significant association with emissions of chlorinated solvents, and these associations tended to be weak with the ORs ranging from 1.06 to 1.23 (Table 4, data for atrioventricular heart defects and anomalous venous return not shown). The only associations noted with limb defects included perchloroethylene with transverse limb deficiencies (aOR 1.21, 95% CI 1.01, 1.45) (Table 5) and any type of chlorinated solvent (exposure risk values summed for 14 chemicals) with lower limb deficiencies (aOR 1.21, 95% CI 1.00, 1.45, data not shown).

**Table 4:** **Maternal residential proximity to air emissions of chlorinated solvents and congenital heart defects, Texas, 1996-2008**

Type of chlorinated solvent	***Exposure risk value >0*** ^***a***^													
	Controls		Conotruncal heart defects		Adjusted odds ratio ^b^(95% CI)		Obstructive heart defects		Adjusted odds ratio ^b^(95% CI)		Septal heart defects		Adjusted odds ratio ^b^(95% CI)	
	***n***	%	***n***	%			***n***	%			***n***	%		
Any type	63599	26.2	941	28.5	1.05	(0.96, 1.14)	912	26.9	1.02	(0.93, 1.11)	11325	26.4	1.06	(1.04, 1.09)
Carbon tetrachloride	4568	1.9	76	2.3	1.09	(0.86, 1.39)	82	2.4	1.16	(0.92, 1.46)	867	2.0	1.13	(1.04, 1.22)
Chloroform	3985	1.6	60	1.8	0.97	(0.74, 1.27)	70	2.1	1.13	(0.88, 1.45)	739	1.7	1.10	(1.01, 1.19)
1,1-dichloroethane	1847	0.8	30	0.9	1.10	(0.76, 1.59)	34	1.0	1.17	(0.83, 1.66)	387	0.9	1.23	(1.10, 1.37)
1,2-dichloroethane	6637	2.7	102	3.1	1.00	(0.81, 1.24)	106	3.1	1.03	(0.83, 1.26)	1184	2.8	1.06	(0.99, 1.13)
1,2-dichloroethylene	2026	0.8	34	1.0	1.13	(0.80, 1.60)	38	1.1	1.20	(0.86, 1.66)	409	1.0	1.19	(1.06, 1.32)
Ethyl chloride	5271	2.2	82	2.5	1.02	(0.81, 1.28)	98	2.9	1.23	(0.99, 1.51)	1002	2.3	1.13	(1.05, 1.21)
Methyl chloroform	5294	2.2	78	2.4	0.97	(0.77, 1.23)	93	2.7	1.14	(0.92, 1.41)	865	2.0	1.01	(0.94, 1.09)
Methylene chloride	38448	15.9	583	17.6	1.04	(0.95, 1.15)	556	16.4	1.02	(0.92, 1.12)	6266	14.6	0.95	(0.92, 0.98)
Perchloroethylene	29854	12.3	426	12.9	1.03	(0.92, 1.15)	413	12.2	1.01	(0.90, 1.12)	5308	12.4	1.02	(0.99, 1.06)
Propylene dichloride	1518	0.6	19	0.6	0.82	(0.52, 1.29)	33	1.0	1.39	(0.98, 1.99)	316	0.7	1.21	(1.07, 1.38)
Tetrachloroethane	1955	0.8	27	0.8	0.93	(0.63, 1.37)	33	1.0	1.09	(0.77, 1.56)	387	0.9	1.14	(1.02, 1.28)
1,1,2-trichloroethane	2561	1.1	40	1.2	1.03	(0.75, 1.42)	48	1.4	1.19	(0.88, 1.59)	487	1.1	1.12	(1.01, 1.24)
Trichloroethylene	28547	11.8	409	12.4	0.98	(0.87, 1.10)	412	12.1	1.03	(0.92, 1.15)	5074	11.8	1.06	(1.02, 1.10)
1,2,3-trichloropropane	2847	1.2	44	1.3	1.01	(0.74, 1.37)	48	1.4	1.06	(0.79, 1.42)	542	1.3	1.13	(1.02, 1.24)

^a^Exposure risk value based on residential proximity to source(s) of air emissions and estimated pounds of chemical emitted annually.

^b^Adjusted for birth year and maternal age, education, race/ethnicity, and public health region of residence (referent group: exposure risk value = 0).

**Table 5:** **Maternal residential proximity to air emissions of chlorinated solvents and limb deficiency defects, Texas, 1996-2008**

Type of chlorinated solvent	***Exposure risk value >0*** ^***a***^													
	Controls		Any type of limb deficiency		Adjusted odds ratio ^b^(95% CI)		Longitudinal limb deficiency		Adjusted odds ratio ^b^(95% CI)		Transverse limb deficiency		Adjusted odds ratio ^b^(95% CI)	
	***n***	%	***n***	%			***n***	%			***n***	%		
Any type	63599	26.2	568	28.7	1.05	(0.94, 1.17)	252	29.3	0.97	(0.82, 1.14)	334	27.7	1.09	(0.94, 1.26)
Carbon tetrachloride	4568	1.9	39	2.0	1.07	(0.77, 1.49)	17	2.0	0.97	(0.59, 1.61)	21	1.7	1.01	(0.64, 1.58)
Chloroform	3985	1.6	37	1.9	1.11	(0.79, 1.57)	16	1.9	1.04	(0.62, 1.74)	20	1.7	1.03	(0.65, 1.63)
1,1-dichloroethane	1847	0.8	14	0.7	1.00	(0.59, 1.71)	7	0.8	1.09	(0.51, 2.32)	7	0.6	0.87	(0.41, 1.85)
1,2-dichloroethane	6637	2.7	52	2.6	0.98	(0.73, 1.32)	24	2.8	0.95	(0.62, 1.46)	29	2.4	0.96	(0.65, 1.42)
1,2-dichloroethylene	2026	0.8	17	0.9	1.09	(0.67, 1.78)	8	0.9	1.11	(0.54, 2.26)	8	0.7	0.89	(0.44, 1.80)
Ethyl chloride	5271	2.2	43	2.2	1.00	(0.73, 1.37)	20	2.3	1.01	(0.64, 1.61)	23	1.9	0.92	(0.60, 1.41)
Methyl chloroform	5294	2.2	46	2.3	0.94	(0.69, 1.27)	23	2.7	0.97	(0.63, 1.48)	23	1.9	0.82	(0.54, 1.26)
Methylene chloride	38448	15.9	361	18.2	1.06	(0.94, 1.20)	164	19.1	1.01	(0.84, 1.22)	204	16.9	1.04	(0.88, 1.23)
Perchloroethylene	29854	12.3	272	13.7	1.10	(0.96, 1.26)	113	13.2	0.95	(0.77, 1.17)	167	13.9	1.21	(1.01, 1.45)
Propylene dichloride	1518	0.6	13	0.7	1.15	(0.66, 2.00)	6	0.7	1.13	(0.50, 2.56)	6	0.5	0.93	(0.41, 2.09)
Tetrachloroethane	1955	0.8	14	0.7	0.99	(0.58, 1.69)	6	0.7	0.93	(0.41, 2.10)	7	0.6	0.86	(0.40, 1.83)
1,1,2-trichloroethane	2561	1.1	18	0.9	0.91	(0.56, 1.46)	8	0.9	0.86	(0.42, 1.75)	9	0.7	0.79	(0.40, 1.54)
Trichloroethylene	28547	11.8	257	13.0	1.01	(0.88, 1.17)	119	13.9	1.00	(0.81, 1.24)	149	12.4	1.04	(0.86, 1.26)
1,2,3-trichloropropane	2847	1.2	24	1.2	1.10	(0.72, 1.67)	11	1.3	1.08	(0.58, 1.99)	12	1.0	0.95	(0.53, 1.71)

^a^Exposure risk value based on residential proximity to source(s) of air emissions and estimated pounds of chemical emitted annually.

^b^Adjusted for birth year and maternal age, education, race/ethnicity, and public health region of residence (referent group: exposure risk value = 0).

Tables 6, 7, and 8 display results of analyses of chlorinated solvents with spina bifida, cleft palate alone, and septal heart defects, respectively, in which exposure risk values were divided into four groups (zero, low, middle, high) based on the control-mother’s distributions. Significant linear trends were noted between the emissions of several chlorinated solvents and spina bifida, including chloroform; 1,1-dichloroethane; 1,2-dichloroethane; ethyl chloride; methyl chloride; tetrachloroethane; 1,1,2-trichloroethane; and 1,2,3-trichloropropane (Table 6). The strongest linear trends were noted with 1,2-dichloroethane (aOR for spina bifida in the fourth quartile: 1.85, 95% CI 1.21, 2.84) and with 1,2,3-trichloropropane (aOR in the fourth quartile: 2.62, 95% CI 1.55, 4.46). Several of the trends were not monotonic in that the highest odds ratios were noted in the second quartile versus the fourth quartile.

**Table 6:** **Intensity of maternal residential exposure index for selected chlorinated solvents and spina bifida in offspring**

Type of chlorinated solvent	Intensity of exposure risk value ^a^	Spina bifida cases		Controls		Adjusted odds ratio ^b^	95% confidence interval	***p-***value for trend
		***n***	%	***n***	%			
Carbon tetrachloride	0.00	1269	96.6	237857	98.1	1.00	Referent	0.059
	0.01-189.30	18	1.4	1531	0.6	2.08	1.29, 3.37	
	189.31-2342.12	14	1.1	1531	0.6	1.53	0.89, 2.64	
	>2342.12	12	0.9	1506	0.6	1.19	0.66, 2.14	
Chloroform	0.00	1276	97.2	238440	98.4	1.00	Referent	0.027
	0.01-42.27	14	1.1	1331	0.5	1.74	1.02, 2.99	
	42.28-1490.26	9	0.7	1331	0.5	1.23	0.63, 2.40	
	>1490.26	14	1.1	1323	0.5	1.66	0.94, 2.91	
1,1-dichloroethane	0.00	1294	98.6	240578	99.2	1.00	Referent	0.046
	0.01-0.53	8	0.6	618	0.3	2.18	1.07, 4.43	
	0.54-6.59	4	0.3	613	0.3	0.94	0.35, 2.53	
	>6.59	7	0.5	616	0.3	2.14	1.00, 4.57	
1,2-dichloroethane	0.00	1248	95.0	235788	97.3	1.00	Referent	< 0.0001
	0.01-5.85	17	1.3	2197	0.9	1.24	0.76, 2.05	
	5.86-221.92	24	1.8	2215	0.9	1.83	1.20, 2.78	
	>221.92	24	1.8	2225	0.9	1.85	1.21, 2.84	
1,2-dichloroethylene	0.00	1293	98.5	240399	99.2	1.00	Referent	0.065
	0.01-0.48	6	0.5	676	0.3	1.51	0.67, 3.41	
	0.49-7.59	8	0.6	677	0.3	1.79	0.88, 3.63	
	>7.59	6	0.5	673	0.3	1.49	0.66, 3.36	
Ethyl chloride	0.00	1263	96.2	237154	97.8	1.00	Referent	0.003
	0.01-12.11	17	1.3	1761	0.7	1.71	1.04, 2.81	
	12.12-98.74	12	0.9	1764	0.7	1.17	0.65, 2.08	
	>98.74	21	1.6	1746	0.7	1.87	1.19, 2.92	
Methyl chloroform	0.00	1256	95.7	237131	97.8	1.00	Referent	0.002
	0.01-5.02	21	1.6	1751	0.7	1.67	1.07, 2.61	
	5.03-198.09	12	0.9	1778	0.7	1.08	0.60, 1.92	
	>198.09	24	1.8	1765	0.7	1.90	1.23, 2.94	
Tetrachloroethane	0.00	1293	98.5	240470	99.2	1.00	Referent	0.033
	0.01-0.18	7	0.5	653	0.3	1.88	0.88, 4.01	
	0.19-1.69	7	0.5	653	0.3	1.83	0.86, 3.92	
	>1.69	6	0.5	649	0.3	1.62	0.72, 3.66	
1,1,2-trichloroethane	0.00	1283	97.7	239864	98.9	1.00	Referent	0.026
	0.01-0.45	15	1.1	853	0.4	3.01	1.77, 5.10	
	0.46-1.78	8	0.6	855	0.4	1.46	0.72, 2.96	
	>1.78	7	0.5	853	0.4	1.40	0.66, 2.98	
1,2,3-trichloropropane	0.00	1282	97.6	239578	98.8	1.00	Referent	< 0.0001
	0.01-16.45	8	0.6	950	0.4	1.31	0.65, 2.68	
	16.46-132.51	8	0.6	947	0.4	1.42	0.70, 2.88	
	>132.51	15	1.1	950	0.4	2.62	1.55, 4.46	

^a^Exposure risk value based on maternal residential proximity to source(s) of air emissions and estimated pounds of chemical emitted annually.

^b^Adjusted for birth year and maternal race/ethnicity, age, education, and public health region.

**Table 7:** **Intensity of maternal residential exposure index for selected chlorinated solvents and isolated cleft palate in offspring**

Type of chlorinated solvent	Intensity of exposure risk value ^a^	Cleft palate cases		Controls		Adjusted odds ratio ^b^	95% confidence interval	***p-***value for trend
		***n***	%	***n***	%			
Propylene dichloride	0.00	1148	98.7	240907	99.4	1.00	Referent	0.031
	0.01-0.02	5	0.4	508	0.2	1.95	0.80, 4.76	
	0.03-17.70	3	0.3	506	0.2	1.03	0.33, 3.25	
	>17.70	7	0.6	504	0.2	2.32	1.09, 4.95	
Tetrachloroethane	0.00	1146	98.5	240470	99.2	1.00	Referent	0.010
	0.01-0.18	3	0.3	653	0.3	0.90	0.29, 2.82	
	0.19-1.69	4	0.3	653	0.3	1.14	0.42, 3.08	
	>1.69	10	0.9	649	0.3	2.66	1.40, 5.04	
1,1,2-trichloroethane	0.00	1141	98.1	239864	98.9	1.00	Referent	0.059
	0.01-0.45	8	0.7	853	0.4	1.66	0.82, 3.37	
	0.46-1.78	4	0.3	855	0.4	0.84	0.31, 2.27	
	>1.78	10	0.9	853	0.4	1.99	1.05, 3.76	
1,2,3-trichloropropane	0.00	1139	97.9	239578	98.8	1.00	Referent	0.083
	0.01-16.45	10	0.9	950	0.4	1.77	0.94, 3.37	
	16.46-132.51	4	0.3	947	0.4	0.74	0.28, 2.00	
	>132.51	10	0.9	950	0.4	1.91	1.01, 3.63	

^a^Exposure risk value based on maternal residential proximity to source(s) of air emissions and estimated pounds of chemical emitted annually.

^b^Adjusted for birth year and maternal race/ethnicity, age, education, and public health region.

**Table 8:** **Intensity of maternal residential exposure index for selected chlorinated solvents and septal heart defects in offspring**

Type of chlorinated solvent	Intensity of exposure risk value ^a^	Septal heart defect cases		Controls		Adjusted odds ratio ^b^	95% confidence interval	***p-***value for trend
		***n***	%	***n***	%			
Any type	0.00	31536	73.6	178826	73.8	1.00	Referent	0.035
	0.01-296.24	4196	9.8	21301	8.8	1.14	1.10, 1.18	
	296.25-3457.84	3542	8.3	21191	8.7	1.00	0.96, 1.04	
	>3457.84	3587	8.4	21107	8.7	1.03	0.99, 1.08	
Carbon tetrachloride	0.00	41994	98.0	237857	98.1	1.00	Referent	< 0.0001
	0.01-189.30	271	0.6	1531	0.6	1.03	0.90, 1.18	
	189.31-2342.12	301	0.7	1531	0.6	1.14	1.01, 1.30	
	>2342.12	295	0.7	1506	0.6	1.21	1.07, 1.38	
1,1-dichloroethane	0.00	42474	99.1	240578	99.2	1.00	Referent	0.003
	0.01-0.53	139	0.3	618	0.3	1.32	1.09, 1.59	
	0.54-6.59	121	0.3	613	0.3	1.18	0.97, 1.44	
	>6.59	127	0.3	616	0.3	1.18	0.97, 1.43	
1,2-dichloroethane	0.00	41677	97.2	235788	97.3	1.00	Referent	0.007
	0.01-5.85	350	0.8	2197	0.9	0.96	0.86, 1.08	
	5.86-221.92	384	0.9	2215	0.9	1.03	0.92, 1.15	
	>221.92	450	1.0	2225	0.9	1.19	1.07, 1.32	
1,2-dichloroethylene	0.00	42452	99.0	240399	99.2	1.00	Referent	0.079
	0.01-0.48	165	0.4	676	0.3	1.42	1.20, 1.69	
	0.49-7.59	127	0.3	677	0.3	1.11	0.92, 1.35	
	>7.59	117	0.3	673	0.3	1.02	0.84, 1.24	
Ethyl chloride	0.00	41859	97.7	237154	97.8	1.00	Referent	0.004
	0.01-12.11	335	0.8	1761	0.7	1.11	0.98, 1.25	
	12.12-98.74	360	0.8	1764	0.7	1.19	1.06, 1.34	
	>98.74	307	0.7	1746	0.7	1.08	0.95, 1.22	
Perchloroethylene	0.00	37553	87.6	212571	87.7	1.00	Referent	0.045
	0.01-41.59	2131	5.0	9990	4.1	1.19	1.13, 1.25	
	41.60-221.22	1605	3.7	9959	4.1	0.94	0.89, 0.99	
	>221.22	1572	3.7	9905	4.1	0.92	0.87, 0.97	
Propylene dichloride	0.00	42545	99.3	240907	99.4	1.00	Referent	0.143
	0.01-0.02	135	0.3	508	0.2	1.55	1.28, 1.88	
	0.03-17.70	101	0.2	506	0.2	1.15	0.93, 1.43	
	>17.70	80	0.2	504	0.2	0.94	0.74, 1.19	
Tetrachloroethane	0.00	42474	99.1	240470	99.2	1.00	Referent	0.113
	0.01-0.18	149	0.3	653	0.3	1.32	1.10, 1.58	
	0.19-1.69	114	0.3	653	0.3	1.00	0.82, 1.23	
	>1.69	124	0.3	649	0.3	1.11	0.91, 1.24	
1,1,2-trichloroethane	0.00	42374	98.9	239864	98.9	1.00	Referent	0.031
	0.01-0.45	148	0.3	853	0.4	1.04	0.87, 1.24	
	0.46-1.78	181	0.4	855	0.4	1.25	1.06, 1.47	
	>1.78	158	0.4	853	0.4	1.08	0.91, 1.28	
Trichloroethylene	0.00	37787	88.2	213878	88.2	1.00	Referent	0.002
	0.01-56.69	1718	4.0	9539	3.9	1.07	1.01, 1.13	
	56.70-284.39	1611	3.8	9485	3.9	1.02	0.96, 1.08	
	>284.39	1745	4.1	9523	3.9	1.09	1.03, 1.15	
1,2,3-trichloropropane	0.00	42319	98.7	239578	98.8	1.00	Referent	0.033
	0.01-16.45	168	0.4	950	0.4	1.05	0.89, 1.24	
	16.46-132.51	210	0.5	947	0.4	1.31	1.12, 1.52	
	>132.51	164	0.4	950	0.4	1.02	0.86, 1.21	

^a^Exposure risk value based on maternal residential proximity to source(s) of air emissions and estimated pounds of chemical emitted annually.

^b^Adjusted for birth year and maternal race/ethnicity, age, education, and public health region.

Although the trends were not uniformly monotonic, the strongest associations between cleft palate alone and propylene dichloride, tetrachloroethane, 1,1,2-trichloroethane, and 1,2,3-trichloropropane were observed in the highest categories of exposure risk values (Table 7). These trends were statistically significant (p <0.05) for propylene dichloride and tetrachloroethane.

Positive linear trends were observed between septal heart defects and seven types of chlorinated solvents, including carbon tetrachloride; 1,1-dichloroethane; 1,2-dichloroethane; ethyl chloride; 1,1,2-trichloroethane; trichloroethylene; and 1,2,3-trichloropropane (Table 8). These associations tended to be weak with the ORs close to 1.00, even in the highest exposure categories, and not monotonic with increasing intensities of exposure risk values, with the exception of odds ratios in relation to carbon tetrachloride emissions. We noted similar patterns of odds ratios and p-values for trend between categorization of exposure risk values into four (Table 8) or seven groups (data not shown), with the exception of all chlorinated solvents combined in which p-values for trend were 0.180 versus 0.035 respectively for the seven versus four levels of categorization. Significant linear trends were observed in the association between ethyl chloride emissions and obstructive heart defects (aOR in the highest exposure risk value 1.49, 95% CI 1.09, 2.05) and in the association between methylene chloride and isolated conotruncal heart defects (aOR in the highest exposure risk value 1.56, 95% CI 1.05, 2.32) (data not shown).

Associations between various chlorinated solvents and birth defects in offspring tended to be stronger among mothers 35 years or older than for younger mothers (Table 9). This pattern was most consistently observed with oral cleft defects and these solvents, including associations with any chlorinated solvent (older versus younger, aORs 1.22 versus 0.99, respectively), carbon tetrachloride (aORs 1.66 versus 1.02), 1,1-dichloroethane (aORs 1.49 versus 0.85), 1,2-dichloroethane (aORs 1.50 versus 0.98), 1,2-dichloroethylene (aORs 1.65 versus 0.87), ethyl chloride (aORs 1.66 versus 1.05), methylene chloride (aORs 1.38 versus 1.02), tetrachloroethane (aORs 1.63 versus 0.85), 1,1,2-trichloroethane (aORs 1.78 versus 1.06), trichloroethylene (with cleft lip with or without cleft palate aORs 1.39 versus 1.02), and 1,2,3-trichloropropane (aORs 1.92 versus 1.12).

**Table 9:** **Maternal residential proximity to air emissions of chlorinated solvents and birth defects by maternal age**

Type of chlorinated solvent	Birth defect group	Number (%) with residential exposure risk value >0, adjusted odds ratios (OR), and 95% confidence intervals (CI)							
		Maternal age group							
		< 35 years				35 years or older			
		Number	(%)	OR ^a^	(95% CI)	Number	(%)	OR ^a^	(95% CI)
Any chlorinated solvent	Control births	56735	(26.2)	˗	˗	6864	(26.3)	˗	˗
	Any type of oral cleft defect	1478	(27.3)	0.99	(0.92, 1.06)	240	(30.6)	1.22	(1.03, 1.46)^b^
	Cleft lip with or without cleft palate	934	(26.7)	0.97	(0.89, 1.06)	161	(33.0)	1.37	(1.10, 1.70)^b,c^
	Obstructive heart defects	767	(26.2)	0.98	(0.89, 1.08)	145	(31.0)	1.27	(1.01, 1.58)^b,c^
Carbon tetrachloride	Control births	4195	(1.9)	˗	˗	373	(1.4)	˗	˗
	Any type of neural tube defect	52	(2.6)	1.31	(0.98, 1.75)	10	(4.7)	2.46	(1.23, 4.91)^b,c^
	Spina bifida	37	(3.2)	1.46	(1.03, 2.07)	7	(4.9)	2.49	(1.09, 5.72)^b^
	Any type of oral cleft defect	122	(2.3)	1.02	(0.85, 1.24)	21	(2.7)	1.66	(1.04, 2.65)^b,c^
	Cleft palate alone	47	(2.4)	1.05	(0.77, 1.42)	8	(2.7)	1.76	(0.84, 3.72)
	Cleft lip with or without cleft palate	75	(2.1)	1.01	(0.79, 1.28)	13	(2.7)	1.59	(0.88, 2.86)
Chloroform	Control births	3662	(1.7)	˗	˗	323	(1.2)	˗	˗
	Any type of neural tube defect	45	(2.3)	1.33	(0.97, 1.81)	7	(3.3)	2.09	(0.93, 4.68)
	Spina bifida	32	(2.7)	1.48	(1.02, 2.14)	5	(3.5)	2.16	(0.82, 5.67)
1,1-dichloroethane	Control births	1704	(0.8)	˗	˗	143	(0.5)	˗	˗
	Any type of oral cleft defect	40	(0.7)	0.85	(0.62, 1.17)	7	(0.9)	1.49	(0.69, 3.25)
1,2-dichloroethane	Control births	6033	(2.8)	˗	˗	604	(2.3)	˗	˗
	Any type of neural tube defect	78	(4.0)	1.31	(1.02, 1.69)	7	(3.3)	0.98	(0.44, 2.16)
	Spina bifida	60	(5.1)	1.70	(1.27, 2.28)	5	(3.5)	1.09	(0.42, 2.80)
	Any type of oral cleft defect	168	(3.1)	0.98	(0.83, 1.16)	29	(3.7)	1.50	(1.00, 2.26)^b^
	Cleft palate alone	64	3.3	1.00	(0.77, 1.31)	13	(4.3)	1.93	(1.05, 3.54)^b^
1,2-dichloroethylene	Control births	1862	(0.9)	˗	˗	164	(0.6)	˗	˗
	Any type of oral cleft defect	45	(0.8)	0.87	(0.64, 1.17)	9	(1.1)	1.65	(0.83, 3.30)
	Cleft palate alone	15	(0.8)	0.75	(0.45, 1.26)	5	(1.7)	2.54	(1.00, 6.43)^b,c^
Ethyl chloride	Control births	4820	(2.2)	˗	˗	451	(1.7)	˗	˗
	Any type of neural tube defect	62	(3.2)	1.36	(1.04, 1.77)	8	(3.7)	1.75	(0.83, 3.68)
	Spina bifida	43	(3.7)	1.48	(1.08, 2.05)	7	(4.9)	2.50	(1.11, 5.63)
	Any type of oral cleft defect	144	(2.7)	1.05	(0.88, 1.25)	24	(3.1)	1.66	(1.07, 2.56)^b^
	Cleft palate alone	52	(2.7)	1.01	(0.76, 1.34)	8	(2.7)	1.41	(0.68, 2.94)
	Cleft lip with or without cleft palate	92	(2.6)	1.08	(0.87, 1.34)	16	(3.3)	1.81	(1.06, 3.07)^b^
	Any type of heart defect	1065	(2.4)	1.14	(1.07, 1.23)	143	(1.8)	0.99	(0.81, 1.20)
	Atrioventricular septal defect	43	(3.8)	1.39	(1.01, 1.91)	4	(1.0)	0.58	(0.21, 1.59)^c^
	Obstructive heart defect	92	(3.1)	1.29	(1.04, 1.61)	6	(1.3)	0.63	(0.28, 1.44)
	Septal heart defect	883	(2.4)	1.14	(1.06, 1.23)	119	(1.8)	0.98	(0.79, 1.20)
Methylene chloride	Control births	34553	(16.0)	˗	˗	3895	(14.0)	˗	˗
	Any type of oral cleft defect	942	(17.4)	1.02	(0.94, 1.10)	158	(20.2)	1.38	(1.14, 1.67)^b,c^
	Cleft lip with or without cleft palate	603	(17.3)	1.02	(0.92, 1.12)	107	(21.9)	1.53	(1.21, 1.93)^b,c^
Tetrachloroethane	Control births	1801	(0.8)	˗	˗	154	(0.6)	˗	˗
	Any type of oral cleft defect	41	(0.8)	0.85	(0.62, 1.16)	8	(1.0)	1.63	(0.79, 3.39)
	Cleft lip with or without cleft palate	24	(0.7)	0.79	(0.53, 1.20)	5	(1.0)	1.57	(0.63, 3.93)
1,1,2-trichloroethane	Control births	2354	(1.1)	˗	˗	207	(0.8)	˗	˗
	Any type of oral cleft defect	70	(1.3)	1.06	(0.83, 1.35)	12	(1.5)	1.78	(0.97, 3.25)
	Cleft palate alone	26	(1.4)	1.03	(0.69, 1.53)	5	(1.7)	2.06	(0.82, 5.19)
Trichloroethylene	Control births	25432	(11.8)	˗	˗	3124	(12.0)	˗	˗
	Cleft lip with or without cleft palate	439	(12.6)	1.02	(0.91, 1.14)	78	(16.0)	1.39	(1.06, 1.83)^b,c^
	Any type of heart defect	5067	(11.6)	1.04	(1.00, 1.07)	1025	(12.6)	1.13	(1.04, 1.22)
	Obstructive heart defects	338	(11.6)	0.97	(0.86, 1.10)	74	(15.8)	1.43	(1.08, 1.88)^b,c^
	Septal heart defects	4217	(11.7)	1.04	(1.01, 1.08)	857	(12.7)	1.13	(1.03, 1.23)
1,2,3-trichloropropane	Control births	2602	(1.2)	˗	˗	241	(0.9)	˗	˗
	Any type of oral cleft defect	83	(1.5)	1.12	(0.90, 1.41)	15	(1.9)	1.92	(1.11, 3.32)^b^
	Cleft palate alone	34	(1.8)	1.22	(0.86, 1.74)	6	(2.0)	2.11	(0.90, 4.94)
	Cleft lip with or without cleft palate	49	(1.4)	1.07	(0.79, 1.43)	9	(1.8)	1.80	(0.90, 3.60)

^a^Odds ratios adjusted for year of birth and maternal education, race/ethnicity, and public health region of residence.

^b^Statistically significant additive interaction by maternal age group.

^c^Statistically significant multiplicative interaction by maternal age group.

ORs for neural tube defects in offspring were also higher in older than younger mothers in relation to residential proximity to emissions of carbon tetrachloride (aORs 2.46 versus 1.31, respectively), chloroform (aORs 2.09 versus 1.33), and ethyl chloride (aORs 1.75 versus 1.36). On the other hand, this pattern was reversed for NTDs by maternal age in relation to 1,2-dichloroethane (younger versus older mothers, aORs 1.31 versus 0.98, respectively). With respect to heart defects, associations were stronger for among older than younger women for residential proximity near trichloroethylene (with obstructive heart defects aORs 1.43 versus 0.97) and any chlorinated solvent emissions (with obstructive heart defects aORs 1.27 versus 0.98). In contrast, associations were stronger for younger mothers than older mothers for the relation between ethyl chloride and atrioventricular septal defects, obstructive heart defects, and septal heart defects in offspring. While only one set of associations with higher odds ratios in younger versus older mothers showed significant multiplicative interaction, 16 sets of the stronger associations in older versus younger mothers showed significant additive and/or multiplicative interaction by maternal age. Too few older case-mothers of babies with limb deficiencies were available to assess interaction by maternal age in this birth defect group.

## Discussion

In this large population-based, case–control study, case-mothers (of offspring with neural tube defects, oral cleft defects, and congenital heart defects) were more likely than control-mothers to have higher estimated exposures to industrial air emissions of chlorinated solvents, based on their residential distance to such facilities and reported annual releases. Associations between maternal residential proximity to these emissions and birth defects in offspring tended to be stronger among mothers who were 35 years or older than among younger mothers, especially with oral cleft defects.

Other studies of the relation between maternal occupational and environmental exposures to chlorinated solvents have indicated associations between these solvents and various birth defects. In the NBDPS population, maternal occupational exposure to chlorinated solvents during the periconceptional period was strongly associated with neural tube defects (aOR 1.96, 95% CI 1.34, 2.97), especially spina bifida (aOR 2.26, 95% CI 1.44, 3.53) [12]. Odds ratios for specific chlorinated solvents were not reported, although the prevalence of occupational exposures to chloroform, methylene chloride, perchloroethylene, trichloroethane, and trichloroethylene were all higher among case-mothers of babies with spina bifida than control mothers. In the present study, case-mothers of babies with spina bifida were also more likely than control-mothers to live near industrial emissions of chloroform, methylene chloride, trichloroethane, and trichloroethylene, but the same proportion of case-mothers as control-mothers lived near industrial emissions of perchloroethylene.

In two French study populations, maternal occupational exposures to chlorinated solvents [11] or halogenated aliphatic solvents [22] (also known as alkyl halide solvents of which chlorinated solvents are a subgroup) were associated with oral cleft defects in offspring, specifically with cleft lip with or without cleft palate. These findings were not corroborated in a U.S. study population in which the odds for any oral cleft, cleft palate, and cleft lip with or without cleft palate in offspring in relation to maternal occupational exposures to chlorinated solvents were close to 1.00 [12]. With respect to environmental exposures, case-mothers of babies were oral clefts were not more likely than control- mothers in Texas to live within a mile of an industrial facility with emissions of alkyl halide solvents [16]. In the present study, few associations were noted between maternal proximity to emissions of specific chlorinated solvents and oral clefts, except for cleft palate with propylene dichloride, tetrachloroethane, and 1,1,2-trichloroethane. With stratification by maternal age, however, proximity to chlorinated solvent emissions overall was significantly associated with any oral cleft and specifically with cleft lip with or without cleft palate among mothers 35 years or older. Stronger associations were observed among older mothers between residential proximity to several types of chlorinated solvent emissions and cleft palate alone and cleft lip with or without cleft palate in offspring.

In a study conducted in France in which metabolites of chlorinated solvents were measured in urine, case-mothers of babies with limb malformations were more likely than control mothers to have higher levels of urinary metabolites of tetrachloroethylene (perchloroethylene) and trichloroethylene [13]. We also observed an association between maternal proximity to emissions of perchloroethylene and transverse limb defects in offspring, but the odds ratios were close to 1.0 for associations between trichloroethylene emissions and limb deficiencies in our study.

With respect to heart defects, maternal occupational exposures to chlorinated solvents were associated with perimembranous ventricular septal defects (aOR 1.7, 95% CI 1.0, 2.8) in the National Birth Defects Study population; other associations were noted with d-transposition of the great arteries and aortic stenosis although the 95% confidence intervals were compatible with the null [14]. In a study of maternal residential proximity to industrial facilities and selected birth defects in offspring in Texas, conotruncal heart defects were not associated with maternal residential proximity to facilities reporting air emissions of alkyl halide solvents [17]. In the present study, we also did not observe any associations with conotruncal heart defects with chlorinated solvent emissions, and most of the significant associations with these solvents were restricted to septal heart defects. However, among older mothers, obstructive heart defects in offspring were associated with maternal proximity to emissions of any chlorinated solvents and specifically with emissions of ethyl chloride and trichloroethylene. Older mothers of offspring with any type of heart defect and septal heart defects were also more likely than older control-mothers to live near emissions of trichloroethylene.

Maternal age has been found in other studies to modify associations between maternal prenatal exposures to chemicals and pregnancy outcomes. In a study of trichloroethylene-emitting sites and congenital heart defects in Wisconsin, older case-mothers (38 years or older) of offspring with congenital heart defects were over three times more likely than older control-mothers to live within 1.32 miles of trichloroethylene-emitting sites; no association between congenital heart defects and this residential characteristic was noted among offspring of younger women [15]. Infants of mothers 35 years or older who were exposed to tetrachloroethylene in drinking water had lower mean birth weights and were more likely to be small for gestational age than infants among unexposed women in the same age group in a study conducted at a U.S. Marine Corps base in North Carolina; these patterns were not observed in offspring of younger mothers [23]. The study investigators suggested that advanced maternal age might increase susceptibility of the developing fetus to chemical insults.

In the present study, we used maternal residential addresses at delivery to determine residential proximity to emissions of chlorinated solvents. While maternal address at delivery corresponds to maternal address during the first trimester for most mothers, results from two previous studies of Texas case-mothers and control-mothers who participated in the National Birth Defects Prevention Study indicated that up to 33% of the case-mothers and 31% of the control-mothers changed residence between conception and delivery [24, 25]. Therefore, some mothers in this study may have been misclassified with respect to residential proximity to emissions of chlorinated solvents during the first trimester, the relevant period of morphogenesis of the birth defects included in this study. On the other hand, older mothers in Texas were observed to move less during pregnancy than younger mothers with approximately 15% of the case- and control-mothers, who were 30 years or older, changing residences between conception and delivery [24]. Furthermore, maternal residential movement of mothers in all age categories tended to involve short distances [25] which would have minimal impact on exposure assessment to air pollutants.

Sufficient address information was not available to geocode all maternal addresses to street level, although proportions geocoded to this level included approximately 87% of the control group addresses and 84 to 87% of case group addresses, with the exception of neural tube defects with 69.1% geocoded to the street level. Geocoded and ungeocoded controls and case groups (heart, oral cleft, limb reduction, and neural tube defect groups) had similar distributions by maternal age, but the ungeocoded case groups and controls had higher proportions of mothers who were Hispanic and who had less than a high school education than the respective geocoded groups. Given that the differences were in the same direction and similar in magnitude among case and control groups, it is most likely that a compensating bias occurred (both cases and controls had the bias in the same direction and of similar magnitude). Therefore, we might expect that the exclusion of the ungeocoded cases and controls to have minimal impact on the odds ratios other than reducing their precision due to reduced sample sizes. Approximately 10% of industrial facilities also could not be geocoded because of insufficient and/or inaccurate address information. These missing industries may have led to misclassification of exposure status for some case and control residences, although it is not likely that this misclassification would have been differential with respect to case or control status.

As a proxy for potential exposure to emissions of chlorinated solvents, we used a metric (EWPM) that incorporated residential distance from all TRI facilities within a 10 km radius that reported air emissions of chlorinated solvents and annual amounts of chemicals reported released from each facility. This approach probably introduced some misclassification of exposures. To assess the performance of EWPM in predicting the presence and intensity of chemical air emissions, we examined how well the EWPM exposure metrics correlated with air measurements of chlorinated solvents taken by the Texas Commission of Environmental Quality at 48 monitoring sites in Texas during 2005. The EWPM measurements were positively correlated with air measurements for the six chemicals available for comparison, including carbon tetrachloride, chloroform, 1,1-dichloroethane, methyl chloroform, 1,1,2-trichloroethane, and trichloroethylene, with the strongest correlations noted with carbon tetrachloride and chloroform [unpublished observations, Gong and Zhan]. In addition to the limitation of chemical quantities aggregated into pounds per year, further misclassification could have been introduced if the amounts released per year varied since the first trimester of some pregnancies might have been during the preceding year. Furthermore, length of gestation for fetal deaths and induced terminations (cases) would have been shorter than that for the majority of live births, although approximately 97% of the cases were liveborn and the most vulnerable period for teratogenesis is the first trimester.

While the Texas Birth Defects Registry includes elective terminations in their active surveillance of birth defects, we were unable to include these cases in this study because of missing data from lack of linkage to a vital record. This lack of linkage had the greatest impact on our analyses of the relation between chlorinated solvent emissions and neural tube defects in which 69% of maternal addresses were geocoded to street level and 22% were missing information regarding maternal education, a variable strongly related to maternal residential proximity to industrial facilities along with maternal race/ethnicity [26].

With respect to specific defects, the highest proportion of induced terminations were found with anencephalic cases (39.7%) with percentages less than 5% for the other studied defects with the exception of spina bifida (8.3%) and lower limb reduction defects (5.6%). Given the high proportion of anencephalic cases that could not be analyzed because of missing information, findings related to this defect should be interpreted cautiously because it is not known whether women who undergo induced terminations are more or less likely to live near sources of industrial emissions.

In this study, we were unable to account for recurrence of birth defects in subsequent pregnancies of case-women, a known risk factor for birth defects [27]. Recurrence would most likely introduce bias into the study if it was related to maternal residence as well as prevalence of birth defects. We are not aware of any studies that document a higher or lower likelihood of mothers living near industrial sites with chemical emissions if they have had a previous birth defect-affected pregnancy. On the other hand, while Lie et al. [28] noted less than a 5% recurrence of birth defects in Norwegian women whose first infants had birth defects, women who resided in the same municipality during both pregnancies were 11.6 times more likely than women whose first infant had no defect, to have a second infant with the same defect. In contrast, women who moved to another municipality after the birth of their first infant were 5.1 times more likely to have a second infant with the same defect. The authors concluded that these findings suggested that environmental factors contribute to the familial risk of birth defects and that important environmental teratogens have yet to be discovered.

## Conclusions

The large sample size of this study allowed examination of associations between specific chlorinated solvents and birth defects as well as stratification by maternal age. Furthermore, a refined exposure assessment accounted for multiple facilities and their respective annual quantities of chemical releases, an improvement from previous studies that estimated exposure based on distance to the nearest industrial facility.

Study findings suggest that maternal residential proximity to air emissions of chlorinated solvents are associated with neural tube, oral cleft, and congenital heart defects, especially among offspring of older mothers. To better understand the relation between maternal exposure to chlorinated solvent emissions and birth defects in offspring, we recommend that future studies focus on populations in which air measurements of these chemicals and residential histories during pregnancy are available.

## Abbreviations

aOR: Adjusted odds ratio
CI: Confidence interval
EWPM: Emission Weighted Proximity Model
NBDPS: National Birth Defects Prevention Study
NTD: Neural tube defects
OR: Odds ratio
TBDR: Texas Birth Defect Registry
TCE: Trichloroethylene
TRI: Toxic Release Inventory
USEPA: United States Environmental Protection Agency.
